# Forefronts and hotspots evolution of the nanomaterial application in anti-tumor immunotherapy: a scientometric analysis

**DOI:** 10.1186/s12951-023-02278-3

**Published:** 2024-01-13

**Authors:** Wei Cao, Mengyao Jin, Weiguo Zhou, Kang Yang, Yixian Cheng, Junjie Chen, Guodong Cao, Maoming Xiong, Bo Chen

**Affiliations:** 1 Department of General Surgery, First Affiliated Hospital of Anhui Medical University, Hefei, 230022 People’s Republic of China; 2 Department of General Surgery, Anhui Public Health Clinical Center, Hefei, 230011 People’s Republic of China; 3 Department of Surgery, The People’s Hospital of Hanshan County, Ma’anshan, 238101 People’s Republic of China

**Keywords:** Sceintometrics, Nano-immunotherapy, Anti-tumor, Nanomaterials, Immunotherapy, Dendritic cells, Drug delivery

## Abstract

**Background:**

Tumor immunotherapy can not only eliminate the primary lesion, but also produce long-term immune memory, effectively inhibiting tumor metastasis and recurrence. However, immunotherapy also showed plenty of limitations in clinical practice. In recent years, the combination of nanomaterials and immunotherapy has brought new light for completely eliminating tumors with its fabulous anti-tumor effects and negligible side effects.

**Methods:**

The Core Collection of Web of Science (WOSCC) was used to retrieve and obtain relevant literatures on antitumor nano-immunotherapy since the establishment of the WOSCC. Bibliometrix, VOSviewer, CiteSpace, GraphPad Prism, and Excel were adopted to perform statistical analysis and visualization. The annual output, active institutions, core journals, main authors, keywords, major countries, key documents, and impact factor of the included journals were evaluated.

**Results:**

A total of 443 related studies were enrolled from 2004 to 2022, and the annual growth rate of articles reached an astonishing 16.85%. The leading countries in terms of number of publications were China and the United States. Journal of Controlled Release, Biomaterials, Acta Biomaterialia, Theranostics, Advanced Materials, and ACS Nano were core journals publishing high-quality literature on the latest advances in the field. Articles focused on dendritic cells and drug delivery accounted for a large percentage in this field. Key words such as regulatory T cells, tumor microenvironment, immune checkpoint blockade, drug delivery, photodynamic therapy, photothermal therapy, tumor-associated macrophages were among the hottest themes with high maturity. Dendritic cells, vaccine, and T cells tend to become the popular and emerging research topics in the future.

**Conclusions:**

The combined treatment of nanomaterials and antitumor immunotherapy, namely antitumor nano-immunotherapy has been paid increasing attention. Antitumor nano-immunotherapy is undergoing a transition from simple to complex, from phenotype to mechanism.

**Graphical abstract:**

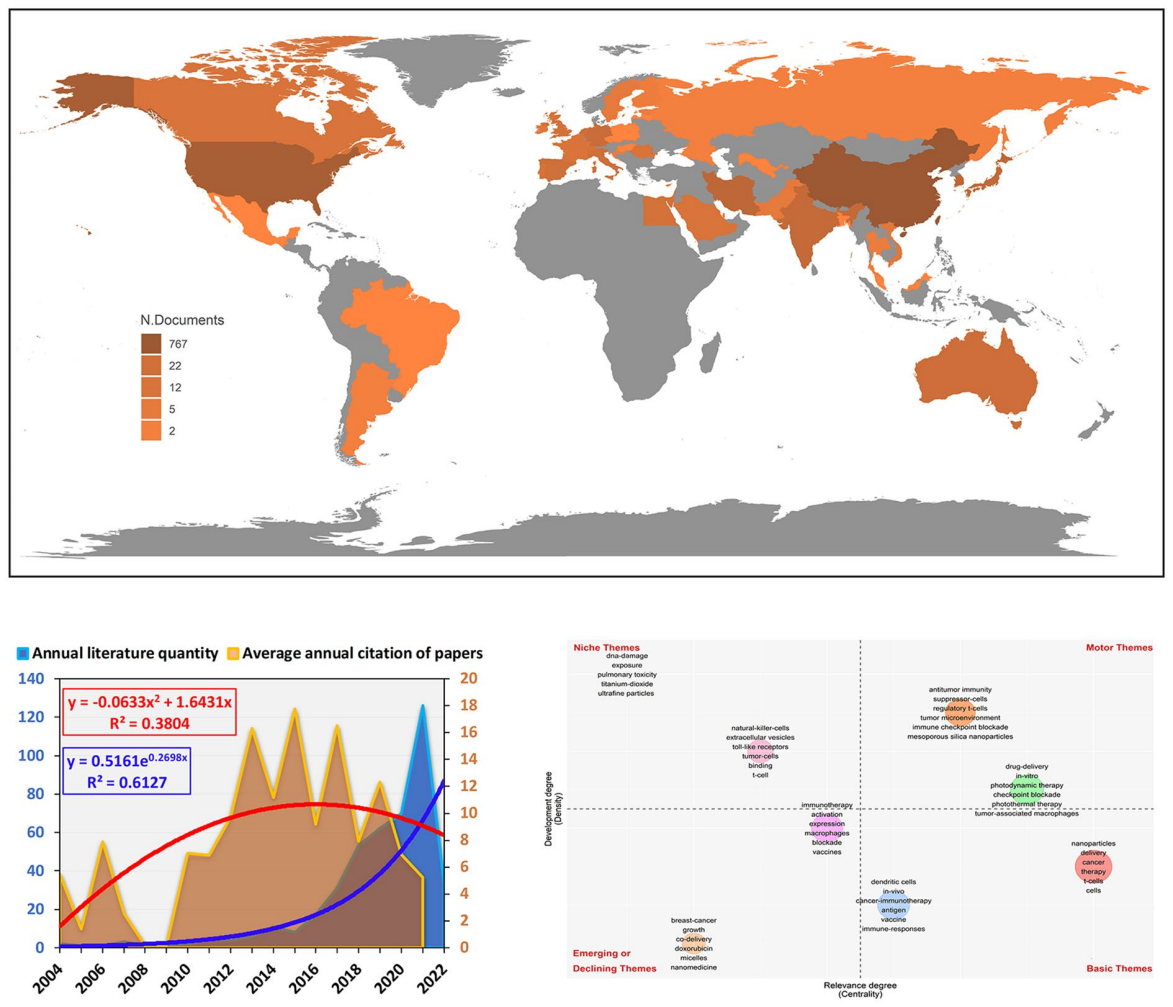

**Supplementary Information:**

The online version contains supplementary material available at 10.1186/s12951-023-02278-3.

## Introduction

A total of 19.3 million new cancer patients were reported worldwide in 2020, with more than 50% dead [1]. Currently, radical surgery is still considered as the most effective treatment for solid tumors. As the early-stage symptoms of many cancers are not typical, numerous patients are already in the advanced stage when diagnosed and miss the timely surgical opportunity. Non-surgical treatments of cancer consist of chemotherapy, radiotherapy, targeted therapy, etc. However, the single treatment method mentioned above always fails to achieve satisfactory therapeutic efficacy [2]. Even though the five-year survival rate of cancer patients has improved in recent years [3], recurrence and metastasis are consistently the number one killer of cancer patients.

In decades, tumor immunotherapy has drawn widespread attention. Different from the traditional treatments, tumor immunotherapy indirectly eliminates tumor cells through regulating immune system rather than directly targeting on tumors. Therefore, it can not only eliminate the primary lesion, but also generate long-term immune memory, thereby inhibiting cancer metastasis and recurrence [4]. So far, more than 3000 immunotherapeutic drugs have been approved by the Food and Drug Administration (FDA) for the treatment of various cancers [5]. Among them, the most famous ones were immune checkpoint blockade (ICB) [6] and chimeric antigen receptor-T cell (CAR-T) [7]. Nonetheless, only a minority of cancer patients showed satisfactory responses to immunotherapy in clinical treatment. Immunotherapy was observed less effective in the majority of the population and even accelerated tumor progression [8, 9]. The following factors were inferred to be responsible for the suboptimal efficacy: 1 The poor immunogenicity of tumors. 2 The low expression level of immunotherapy target. 3 Various immunosuppressive factors in the tumor microenvironment. 4 Inhibition of immune killer cells (such as effector T cells) [4]. Consequently, more and more researchers are seeking for a novel combination treatment system, exploring the possibility to combine immunotherapy with therapies such as radiotherapy, chemotherapy, and immunomodulators [10, 11]. Although these combination therapies enhanced the antitumor efficacy, at the same time the increased incidences of severe side effects were also observed [12]. Notably, the combination of nanomaterials and immunotherapy has brought a new light for completely eliminating tumors with fabulous anti-tumor effects and negligible side effects.

Nanomedicine refers to the application of nanotechnology in medicine. Conventional nanomedicine refers to intravenous injection of materials with a size of about 1–100 nm, which can passively or actively accumulate in pathological areas. The materials or the loaded drugs act at local lesions, realizing precise treatment with lower drug dosage and lighter side effects. The tumor targeting effects of nanomaterials are mainly achieved in two ways, namely passive targeting and active targeting. Passive targeting relies on the enhanced permeability and retention (EPR) effect [13] while active targeting relies on targeting ligands (such as targeting peptides and antibodies) on nanomaterials [14]. Given the promising future and current limitations of immunotherapy, more and more researchers have made efforts on exploring how to apply nanomedicine technology into tumor immunotherapy to create a novel combined therapy. In 1998, researchers discovered [15] that delivery of tumor antigens to antigen presenting cells (APC) through poly-lactide-co-glycolide (PLG) triggered a strong anti-tumor immune response, which protected mice from P815 threat of tumor cells. Similarly, a study by Murthy et al. [16] synthesized an acid-sensitive microgel material, which could be degraded in the acidic phagosome of APC, thereby releasing protein antigens. Although the design of these nanomaterials is relatively rudimentary from today's perspective, it undoubtedly brought new light on nano-immunotherapy for subsequent researchers. At present, nano-immunotherapy is generally achieved through the following three methods [17, 18]: 1 Target and eliminate tumor cells, further causing immunogenic death. 2 Target the tumor immune microenvironment (TIME), such as immune cells (macrophages, dendritic cells, T cells, etc.) or immune-related pathways (such as PD-1/PD-L1, CTLA-4, etc.). 3 Target the peripheral immune system, such as promoting the production of APCs and cytotoxic T cells in lymph nodes and spleen.

Scientometrics uses mathematical and statistical methods to quantitatively analyze overall relevant documents in a certain period of time. Through scientometrics, we can intuitively obtain the development trend in concerned research field, as well as the contributions of various authors, institutions, and countries to the field. More importantly, scientometrics can predict the future development direction of the field. In the past ten years, more and more nanomaterials have been applied in anti-tumor immunotherapy, which activated human autoimmune system through a variety of pathways. Therefore, in this article scientometrics was adopted to further count and analyze the key points of studies in nano-immunotherapy, so that researchers can more intuitively observe the hot spots and prospective development directions of anti-tumor nano-immunotherapy.

## Materials and methods

### Data collection and retrieval strategy

The Core Collection of Web of Science (WOSCC) was used to retrieve and obtain relevant literatures on antitumor nano-immunotherapy since the establishment of the WOSCC. All articles were retrieved on the same day to prevent partial results confusion due to rapid updates of subsequent publications. The search string applied was: (Topic = [“Tumor” OR “Neoplasm” OR “Cancer” OR “Neoplasias” OR “Malignancy”]) AND Topic = “Nano” AND Topic = “Immune”. According to the above retrieval formula, 893 potentially relevant papers were obtained. The following exclusion criteria was adopted to arrive at the final number of records to be analyzed: 1 Literature not related to the subject. 2 Non-English literature. 3 Documents without a complete research process such as conferences and comments. Thereafter, the authors looked through the titles and abstracts and screened out 364 irrelevant records. After reading the full text, authors further screened out 86 articles. Finally, a total of 443 records including 294 articles and 149 reviews were considered for the final analysis. The bibliometric information of 443 articles collected include: title, publication year, author, country/region, affiliation, journal, keywords, keywords plus, number of citations and reference records, abstract, Impact Factor (IF), etc.

### Statistical analysis

The data was exported in plain text format as well as in RIS format. The file in plain text was imported to Biblioshiny for bibliometrix (online website based on Bibliometrix 4.1.0), and the processed excel file including title, publication year, author, country/region, affiliated institution, periodical, keywords, keywords plus, cited times and reference records, abstract and other information was exported for further analysis and interpretations. In addition, the file in RIS format was imported into NoteExpress software, and the file in Excel format (including the IF of the concerned records) was exported. Finally, the final version was obtained after merging the two Excel files. The statistical analysis of this study is based on the comprehensive table (Additional file 6: Table S1).

The above collated data was imported into the R language-based Bibliometrix 4.1.0 package, VOSviewer (version 1.6.18), CiteSpace (version 5.8.R2) and Excel (version 2019) to perform statistical analysis and visualization.

Bibliometrix [19, 20] is a bibliometric statistics and visualization tool based on R language, which has been adopted by more than a thousand bibliometric papers. Strategic diagram is a two-dimensional diagram constructed with the density index as the ordinate and the centrality index as the abscissa. Larger Density index indicates a higher maturity of the topic. Larger Centrality index indicates that the topic is closely related to other topics and that the topic is at the core of all research topics [21, 22].

Vosviewer [23, 24] software was used to make density visualization of keywords co-occurrence. Each point on the map is filled with color according to the density of the elements around the point. The higher the density, the closer the color is to the red; on the contrary, the lower the density, the closer the color is to the blue. Density is positively correlated with the number of elements in the surrounding area and the importance of those elements. CiteSpace [25, 26] software was used for cluster analysis of keywords and time axis view visualization of keywords. In CiteSpace, Modularity Q > 0.3 and Weighted Mean Silhouette > 0.5 indicate that the clustering results are convincing enough.

All radar charts, histograms, line charts and scatter plots were analyzed using Excel 2019. All violin plots were analyzed by GraphPad Prism 8. All heat maps including correlation heat maps were performed using the OmicStudio tools (https://www.omicstudio.cn/tool) [27]. Journals’ Impact Factor was retrieved from the 2020 Journal Citation Reports (JCR). *P* < 0.05 was considered statistically significant.

## Results

### General overview

**Fig. 1:**
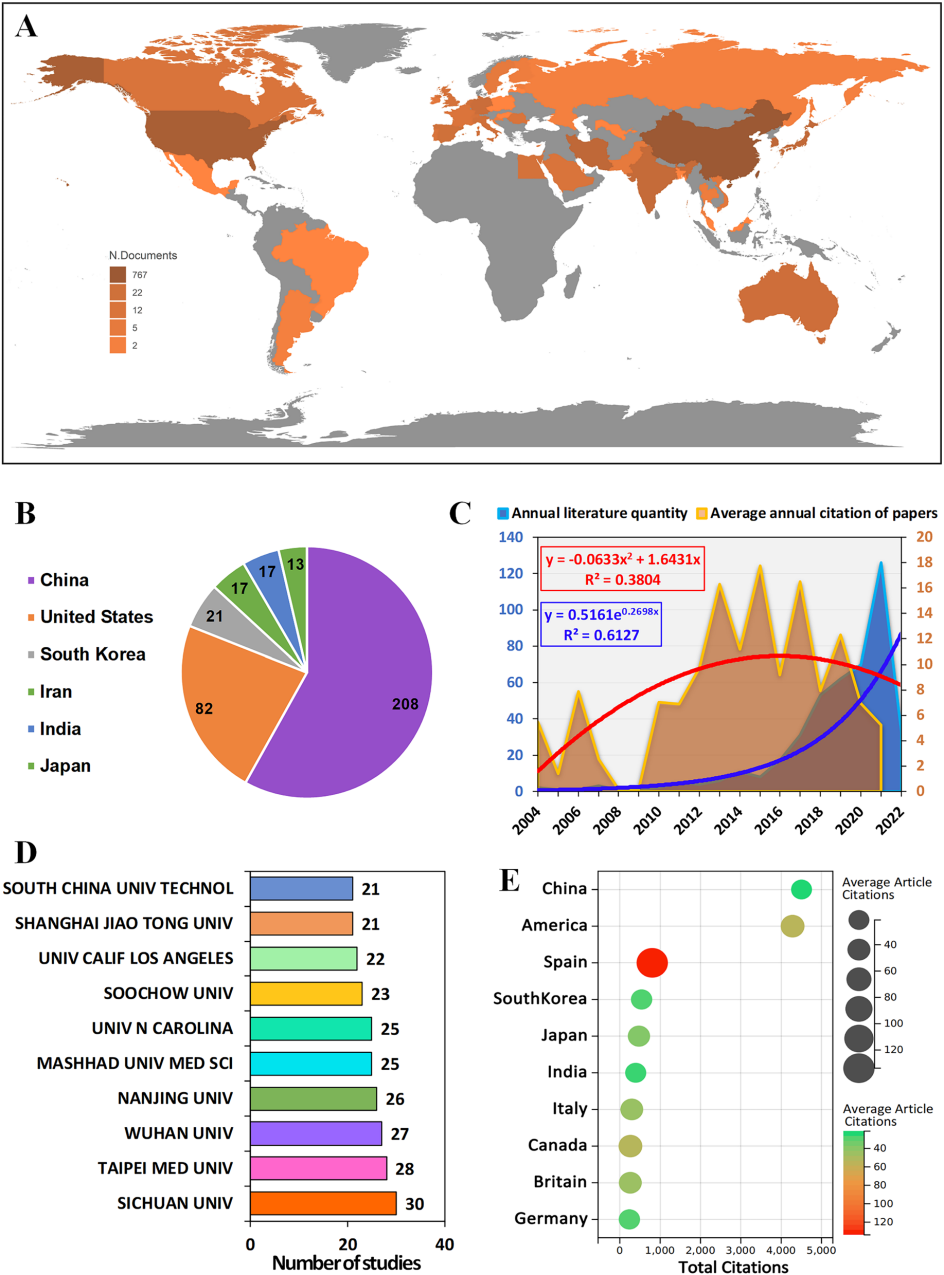
Basic information of included literature. **A** The distribution of countries in terms of publications. **B** The top 6 most productive countries/regions. **C** Annual publications quantity and average annual citation of publications. **D** The number of publications contributed by the top ten institutions. **E** The number of citations from the top ten contributing countries

A total of 34 countries published relevant literature on antitumor nano-immunotherapy (Fig. 1A, Additional file 7: Table S2). The top six countries with the largest number of publications were China (208), the United States (82), South Korea (21), Iran (17), India (17) and Japan (13) (Fig. 1B). The country of relevant documents was based on the country of the corresponding author and the first author. The number of studies on antitumor nano-immunotherapy has grown exponentially in recent years, and we speculated that the growth rate of related literature would still increase in the next few years (Fig. 1C). In addition to the first author and the corresponding author, every author contributed a lot to the paper. Therefore, we completely agreed with your suggestions. We included the entire authors of the article and further counted the total number of authors in each country to evaluate the contribution of the country (Additional file 1: Fig. S1, Additional file 7: Table S3). Additional file 2: Fig. S2 Showed the top six countries contributing to this field. Additional file 3: Fig. S3 showed the cooperation between different countries.

Ten institutions have published more than 20 relevant papers: Sichuan University (Sichuan Province, China, 30), Taipei Medical University (Taiwan Province, China, 28), Wuhan University (Hubei Province, China, 27), Nanjing University (Jiangsu Province, China, 26), North Carolina State University (North Carolina, USA, 25), Mashhad University of Medical Sciences (Reza Khorasan, Iran, 25), Soochow University (Jiangsu, China, 23), University of California, Los Angeles (California, USA, 22), Shanghai Jiaotong University (Shanghai, China, 21), South China University of Technology (Guangdong, China, 21) (Fig. 1D). We also selected the affiliated institution of the first corresponding author for the follow-up analysis (Additional file 4: Table S4). As numerous different institutions may exist in one article, we thought the affiliated institution of the first corresponding author could be most representative. The total number of citations of relevant researches in China (4504) and the United States (4282) were far ahead of other countries. The average number of citations in the Spanish literature was 133.8, ranking firmly in the first place. Meanwhile, the average number of citations the United States was 53.5, and that of China was only 21.2 (Fig. 1E).

**Fig. 2:**
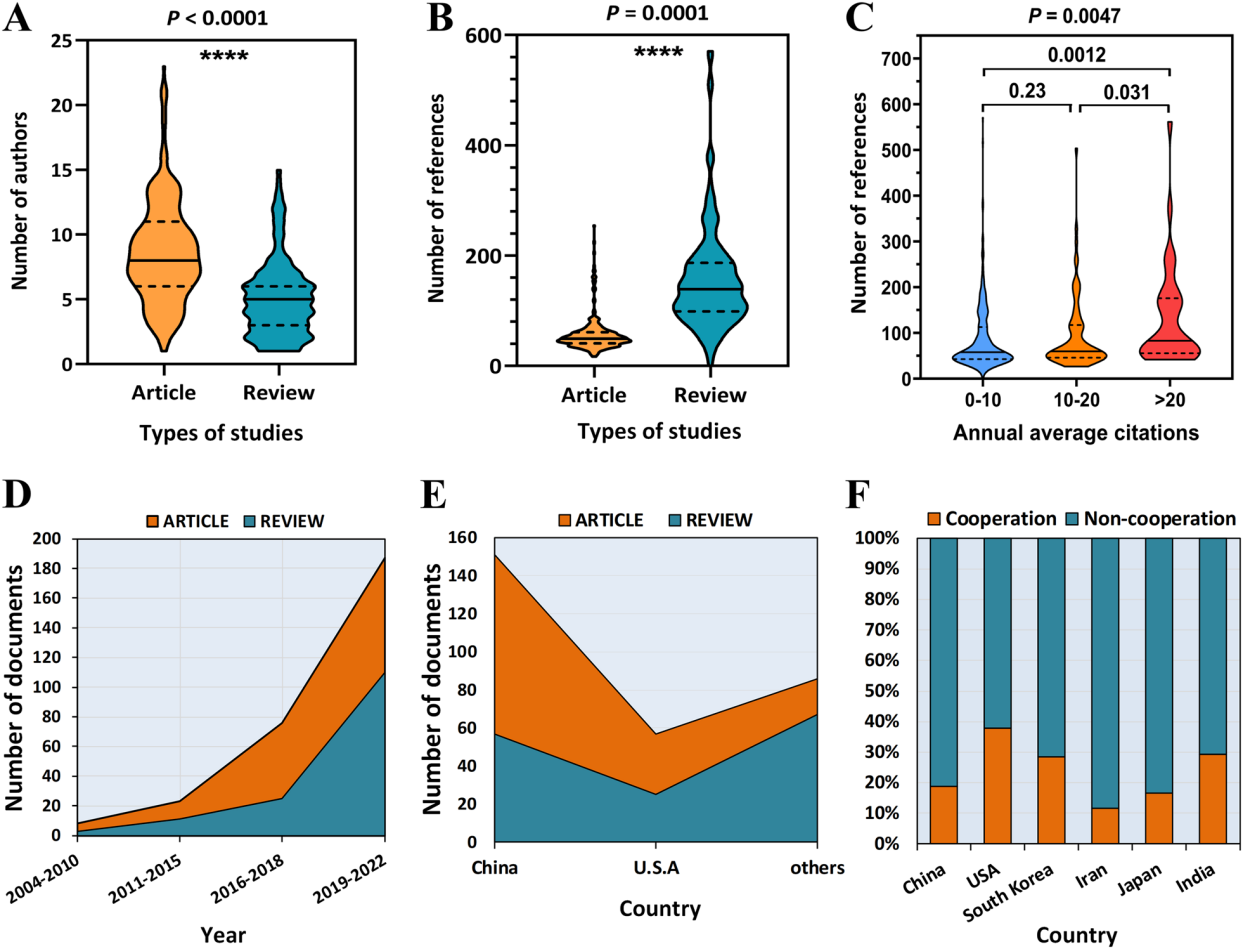
The difference in the number of authors **A** and references **B** between article and review. **C** The relationship between the annual average number of citations and the number of references. **D** The growth curve of different years. **E** The proportion between Articles and reviews between different countries. **F** Differences in the proportion of national cooperation in the literature of different countries

Among all relevant records, the total number of authors of experimental articles (8.61) was significantly larger than that of review literature (5.05) (Fig. 2A). The number of references in the review literatures (156.8) was much greater than that in the experimental papers (56.77) (Fig. 2B). Studies with more than 20 citations had more references (Fig. 2C). Both experimental articles and review articles have increased rapidly in recent years, and the total number and growth rate of experimental articles are greater than that of review literatures (Fig. 2D). In the relevant articles published in China and the United States, the number of experimental articles was about twice that of review literature. However, review literatures accounted for the vast majority of articles published in other countries (Fig. 2E). For the above six countries with the largest number of publications, about 38% of the articles published in the United States were completed by multiple countries, while the proportion of articles published in Iran was only 12% (Fig. 2F).

### Journal correlation analysis

**Fig. 3:**
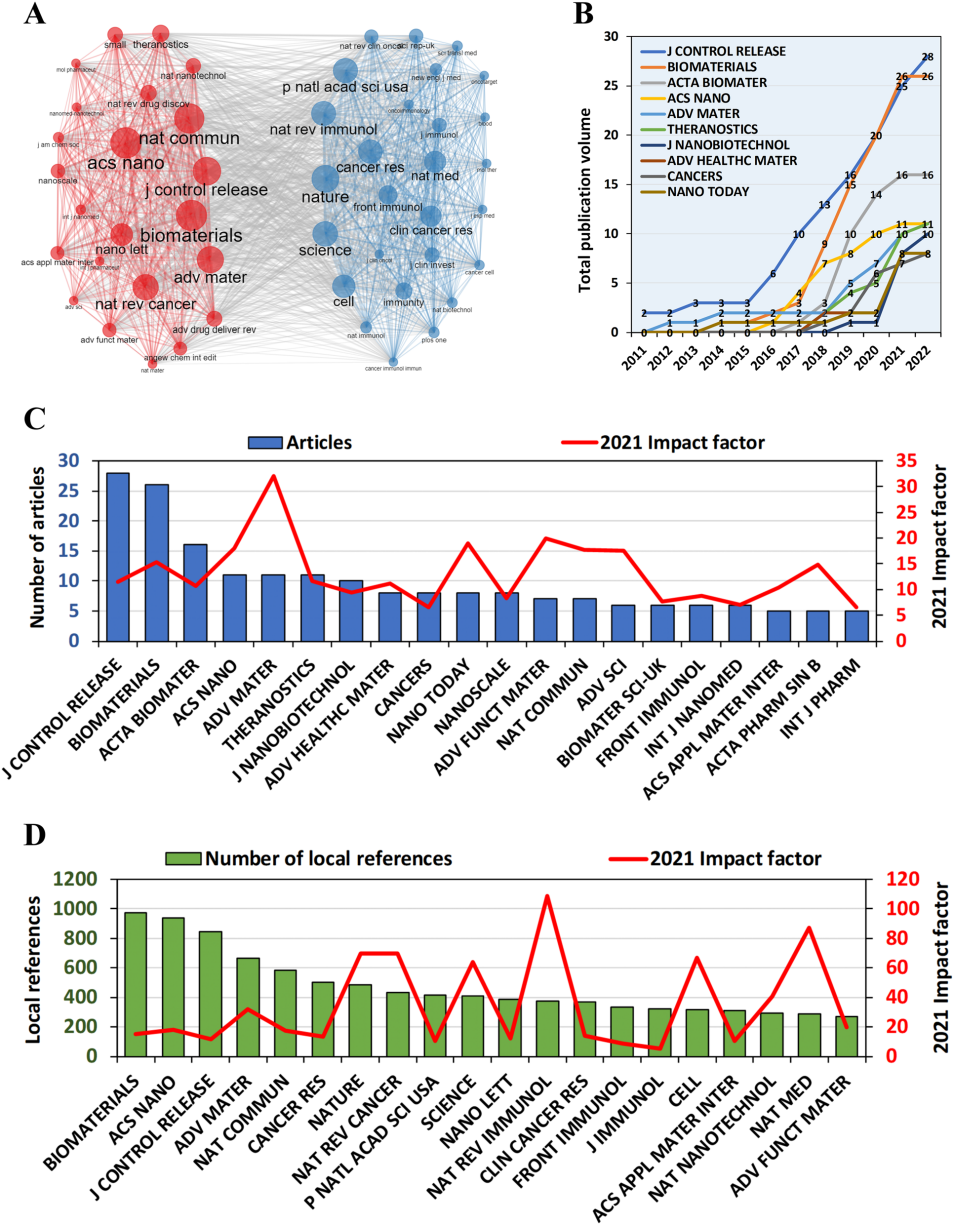
Journal correlation analysis. **A** Co-citation network of References. **B** The line chart of the total number of publications of the top ten journals over time. **C** The top 20 journals with the highest publication volume and their impact factor. **D** The 20 journals with the highest number of local references and their impact factor

The journal co-citation network showed that papers in the field of anti-tumor nano-immunotherapy was mainly published in two types of journals (Fig. 3A). The red colour represents journals of materials science, while the blue parts were mainly medical journals. We found that the co-citations between the two clusters were abundant, which was consistent with the theme of nanomaterials for tumor immunotherapy. In the past five years, the two journals, Journal of Controlled Release and Biomaterials, have seen the fastest growth in the number of articles published in related fields (Fig. 3B). Figure 3C shows that the Journal of Controlled Release published by Netherlands published the highest number of records on the subject (a total of 28 published records, 2021 IF = 11.467), followed by Biomaterials published by Netherlands (26 publications in total, 2021 IF = 15.304), and Acta Biomaterialia published by England (a total of 16 publications, 2021 IF = 10.633). There were three journals with the fourth largest number of publications, each publishing 11 articles, namely Theranostics (2021 IF = 11.6), Advanced Materials (2021 IF = 32.086), and ACS Nano (2021 IF = 18.027). It is worth mentioning that the top 20 journals by publication volume belonged to the Q1 division (2021 JIF quartile), and the impact factor of each of the top-ranked journals was above 10. Therefore, it can be concluded that the idea of antitumor nano-immunotherapy is generally recognized by high-quality journals. On the other hand, the top five journals cited articles under study are: Biomaterials, ACS Nano, Journal of Controlled Release, Advanced Materials, Nature Communications (Fig. 3D). The top 20 journals included many top journals in the industry, such as Nature, Science, Cell, Advanced Materials, Nature Reviews Immunology, Nature Nanotechnology, etc. This reflects that the theoretical foundation of antitumor nano-immunotherapy was solid.

### Author related analysis

**Fig. 4:**
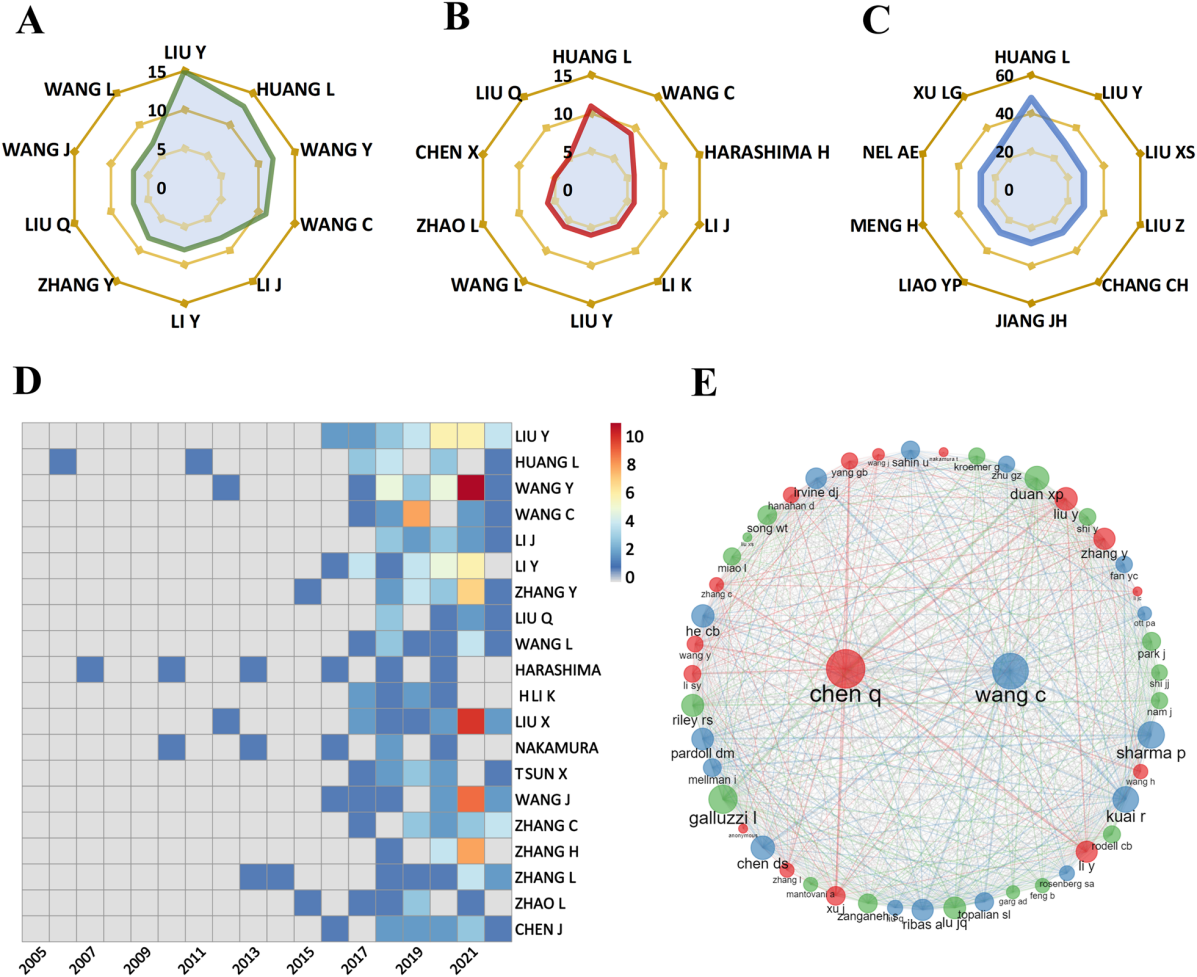
Author related analysis. **A** The top ten authors with the highest number of publications. **B** The top ten authors with the highest H-index. **C** The top ten authors with the highest number of citations from local articles. **D** The heat map of the annual publication volume of the top 20 authors. **E** Co-citation network of References

We calculated the H index and the citations for different authors in articles relevant to this filed, and all analyses were performed only in the 443 included articles. There are many experts and scholars majoring in the field of antitumor nano-immunotherapy. A total of 2571 authors, averaging 7.41 authors per article contributed to records under study. Amongst them, the top 3 authors were LIU Y (15 papers), HUANG L (13 papers), and WANG Y (12 papers) (Fig. 4A). The top three authors with the highest local H-index were HUANG L (11), WANG C (9), and LIU Y (6) (Fig. 4B). The top three authors cited most by local literature were HUANG L (48), LIU Y (30), LIU XS (29) (Fig. 4C). The heat map of the annual publication volume of the top 20 authors is shown in Fig. 6D. The author co-citation network is shown in Fig. 6E. It can be seen that the key authors in the field of nanomaterials applied to tumor immunotherapy included Prof. Yang Liu (Nankai University, China), Prof. Leaf Huang (University of North Carolina at Chapel Hill, USA) and Prof. Chao Wang (Soochow University, China).

### Keywords correlation analysis

**Fig. 5:**
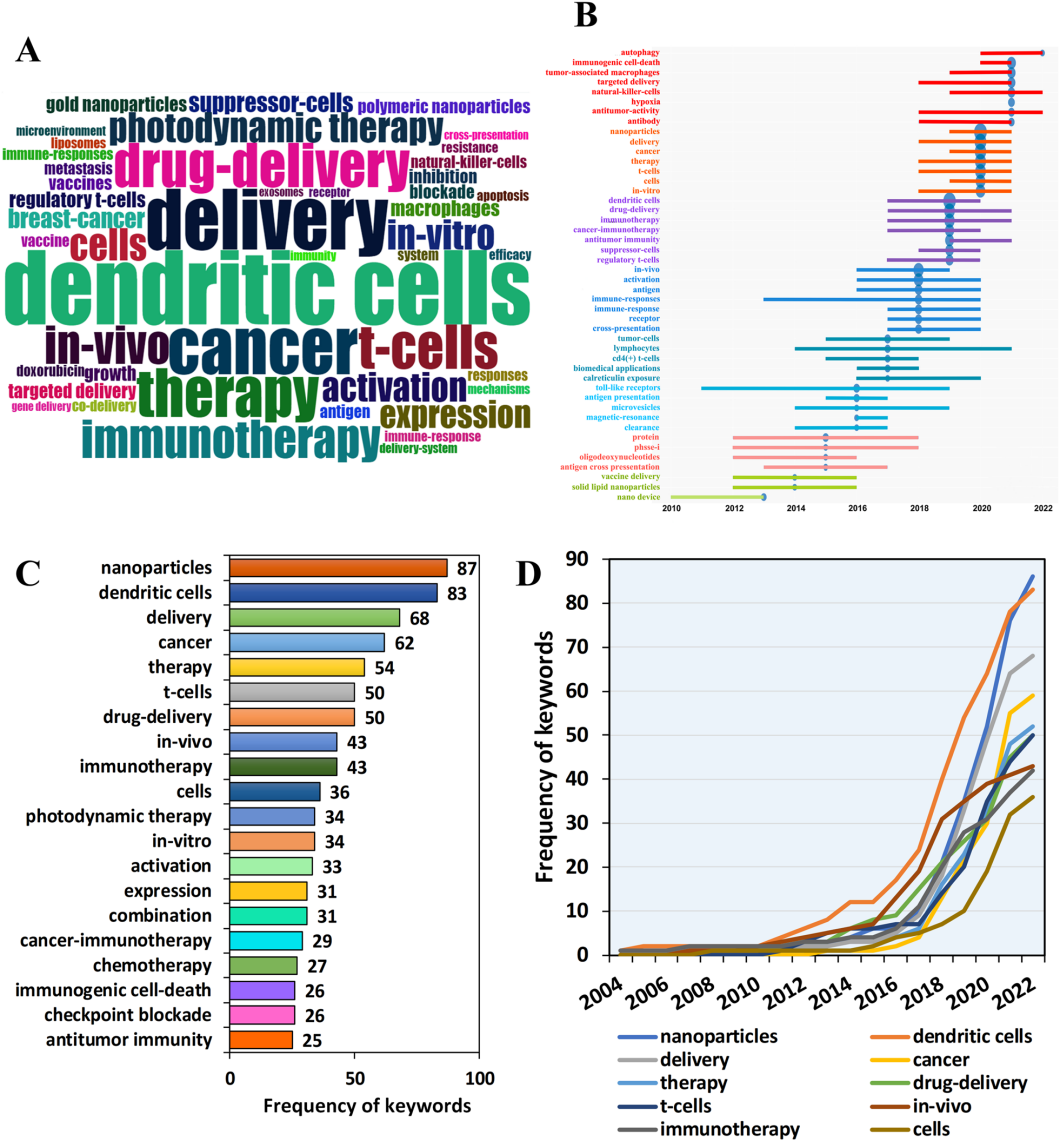
Keywords correlation analysis. **A** Word cloud of keywords. **B** Annual trend chart of keywords changes. **C** The top twenty keywords with the highest frequency. **D** Annual line chart of keywords frequency changes

The cloud map of keywords (Fig. 5A) shows that dendritic cells, delivery, cancer, T cells, immunotherapy, and photodynamic therapy were the key research directions for the application of nanomaterials in tumor immunotherapy. The keywords density map (Additional file 4: Fig. S4) showed that in addition to the above keywords, immune checkpoint blockade, chemotherapy, tumor microenvironment, immune response, etc. were also hot research topics in related fields. The top five keywords with the highest frequency of occurrence are (Fig. 5C): nanoparticles (87), dendritic cells (83), delivery (68), cancer (62), therapy (54). The annual term frequency line chart of keywords included in the literature showed that the usage of above keywords has grown rapidly in recent years (Fig. 5D). Through the correlation heat map (Additional file 5: Fig. S5), we concluded that immunotherapy is most closely related to keywords, such as immune checkpoint blockade (correlation coefficient = 0.71), photodynamic therapy (correlation coefficient = 0.65), photothermal therapy (correlation coefficient = 0.57), T cells (correlation coefficient = 0.58), tumor-associated macrophages (correlation coefficient = 0.52), tumor microenvironment (correlation coefficient = 0.47), immunogenic cell death (correlation coefficient = 0.43), etc. In addition, dendritic cells were closely related to vaccine (correlation coefficient = 0.46), and photodynamic therapy was closely related to checkpoint blockade (correlation coefficient = 0.33).

The annual main keywords evolution chart reveals (Fig. 5B) that the main keywords in 2022 was autophagy; 2021 included immunogenic cell death, tumor-associated macrophages, targeted delivery, natural killer cells, hypoxia, antitumor-activity, antibody and in 2020 included delivery, T cells, etc. The main keywords in 2019 included dendritic cells, drug delivery, regulatory T cells, etc. The earlier keywords included vaccine delivery, antigen cross-presentation, cd4(+) t-cells, etc.

**Fig. 6:**
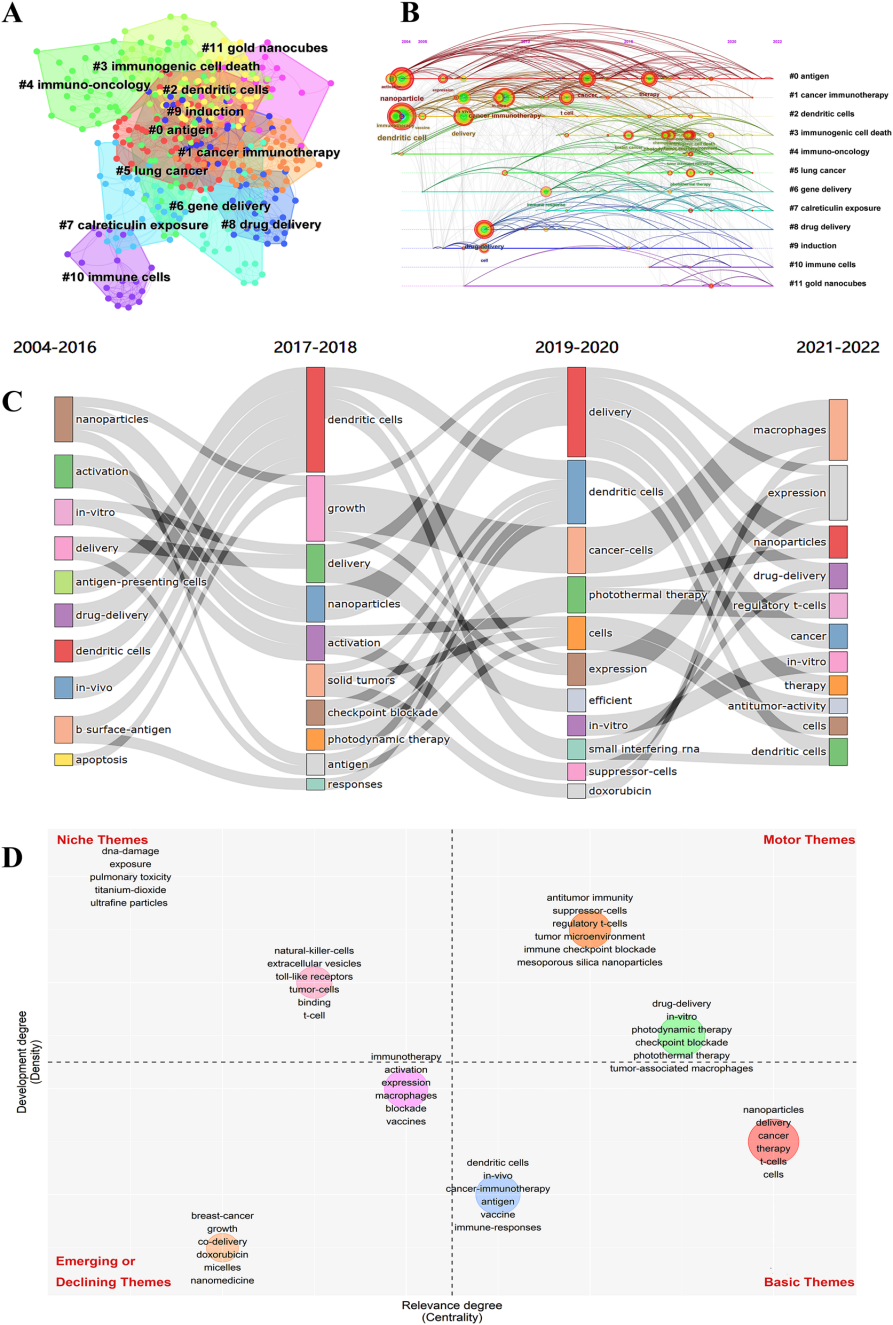
The evolution trend of keywords. **A** Cluster analysis of keywords. **B** Timeline distribution of the top 12 clusters. **C** The changes and internal connections of keywords in different time periods. **D** The Strategic diagram displays the development trend and maturity level of keywords

The co-occurrence analysis of keywords shows (Fig. 6A) that keywords were divided into 12 clusters, represented by different colors. Amongst them, Modularity Q = 0.5624 and Weighted Mean Silhouette = 0.7886 indicated that the clustering results were convincing enough. We found that the representative words of the first three clusters were: antigen, cancer immunotherapy, dendritic cells. Based on the above clustering, we further obtained an evolution timeline of keywords clustering (Fig. 6B). As shown in Fig. 6C, some studies have been enduring in recent years, such as drug-delivery and dendritic cells.

Strategic diagram of the sub-period (Fig. 6D) showed that regulatory T cells, tumor microenvironment, immune checkpoint blockade, drug-delivery, photodynamic therapy, photothermal therapy, tumor-associated macrophages, etc. located in the Motor Themes quadrant, indicating that the above keywords were the core theme with high maturity. In addition, dendritic cells, vaccine, and T cells were located in the Basic Themes quadrant, which demonstrated that the above keywords were important but the current research was not enough, so the above topics may become research hotspots or future development trends.

### Country related analysis

In fact, cooperation among authors of different countries in this field is very common, with the proportion of international cooperation in the included literature being as high as 28.67%. As a representative of developed countries, the United States has cooperated with a number of developed and developing countries, such as China, South Korea, India, Egypt, Saudi Arabia, Argentina, Iran, Israel, Spain, Portugal and so on. The United States and China, as the leaders in this field, have maintained close cooperation with the rest of the world, which is particularly crucial to the common progress of global medicine. In addition, developing countries such as India, Iran, Egypt, Saudi Arabia and Romania also play an increasingly important role in this field. With the joint efforts of both developed and developing countries, this field will move towards a better future.

**Fig. 7:**
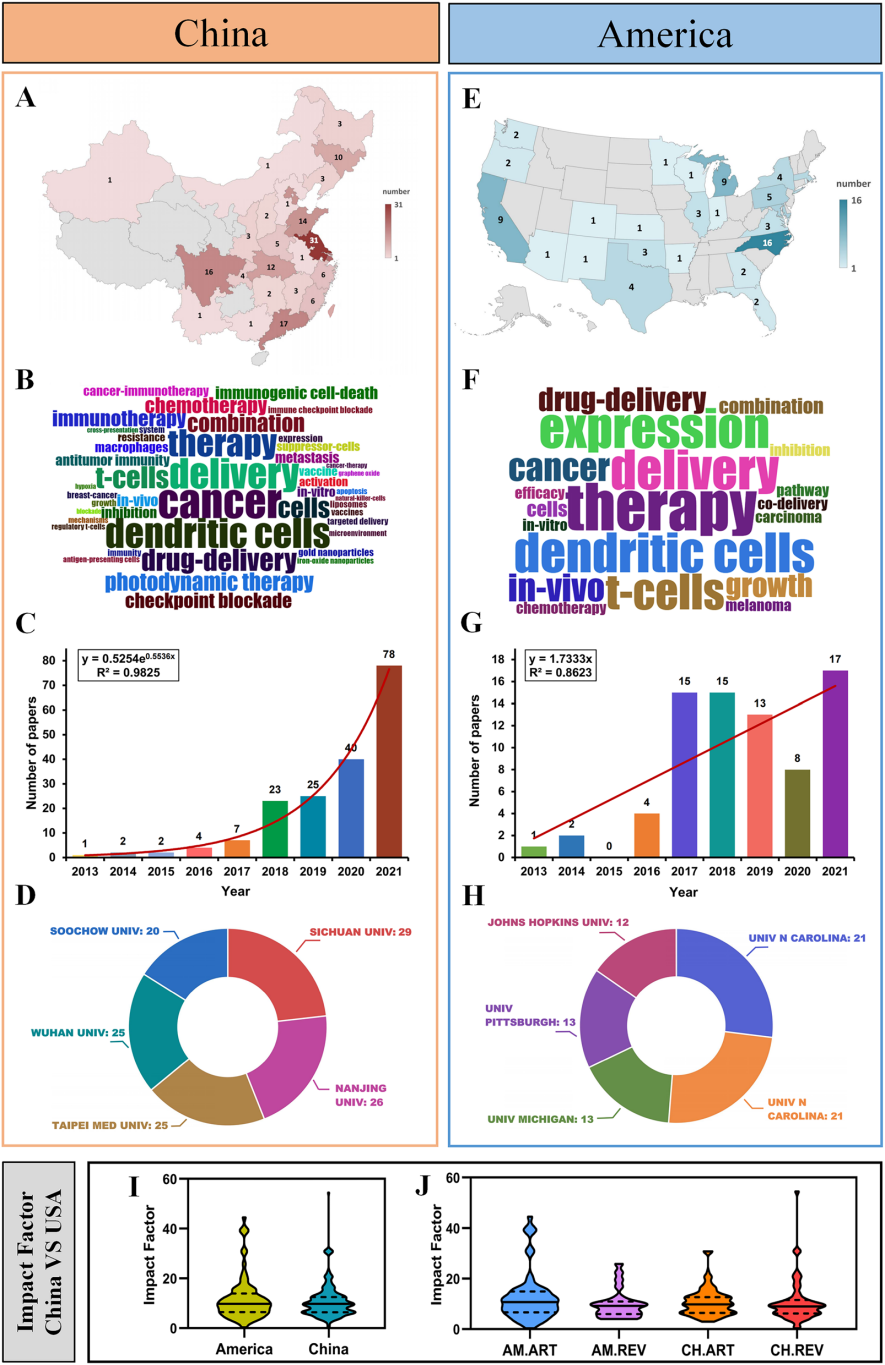
The development differences between China and the United States in this field. The number of publications in different regions of China (**A**) and America (**E**). The word cloud map of China (**B**) and America (**F**). The growth curve of publication volume in China (**C**) and America (**G**). The top five institutions in terms of publication volume in China (**D**) and America (**H**). **I** The differences in impact factor between Chinese and American literature. **J** The differences in impact factor between different types of papers in China and America

The country of the document was based on the country of the corresponding author. If there were corresponding authors affiliated with institutions in different countries, the country of the first author shall prevail. Researches in the field of antitumor nano-immunotherapy were mainly carried out in China and the United States. The rest of the countries published less papers and were not further analyzed. The heat map of the number of papers published by each province in China (Fig. 7A) showed that the provinces of related fields were mainly distributed in the southeastern provinces, of which Jiangsu Province (31), Shanghai (22), and Guangdong Province (17) contributed the most. The heat map of the number of publications by provinces in the United States (Fig. 7E) showed that the publications of related fields were mainly distributed on the east coast, of which North Carolina (16), Michigan (9), and California (9) contributed the most.

In the field of antitumor nano-immunotherapy, research hotspots shared by China and the United States included dendritic cells, delivery, T cells, and combination. There were some unique research hotspots in China: immune checkpoint blockade, photodynamic therapy, and immunogenic cell death. The US-specific research hotspot was expression (Fig. 7B, F).

The number of articles related to China and the United States has grown rapidly in 2022. Among them, the number of publications in China showed an exponential rise between 2013 and 2021, while the United States showed a linear growth (Fig. 7C, G). The top five institutions in China for publishing papers included SICHUAN UNIV, NANJING UNIV, TAIPEI MED UNIV, WUHAN UNIV, and SOOCHOW UNIV. The top five institutions in the United States included UNIV N CAROLINA, UNIV CALIF LOS ANGELES, UNIV MICHIGAN, UNIV PITTSBURGH, and JOHNS HOPKINS UNIV (Fig. 7D, H).

Adopting IF as an evaluation indicator, the quality of papers in China and the United States were similar. The average IF of American articles was 11.84, while the average IF of Chinese articles was 10.74, and there was no significant statistical difference between the two (Fig. 7I). In addition, there was also no statistical difference in IF between review articles and experimental articles in China and the United States (Fig. 7J).

### Correlation analysis of key papers

**Fig. 8:**
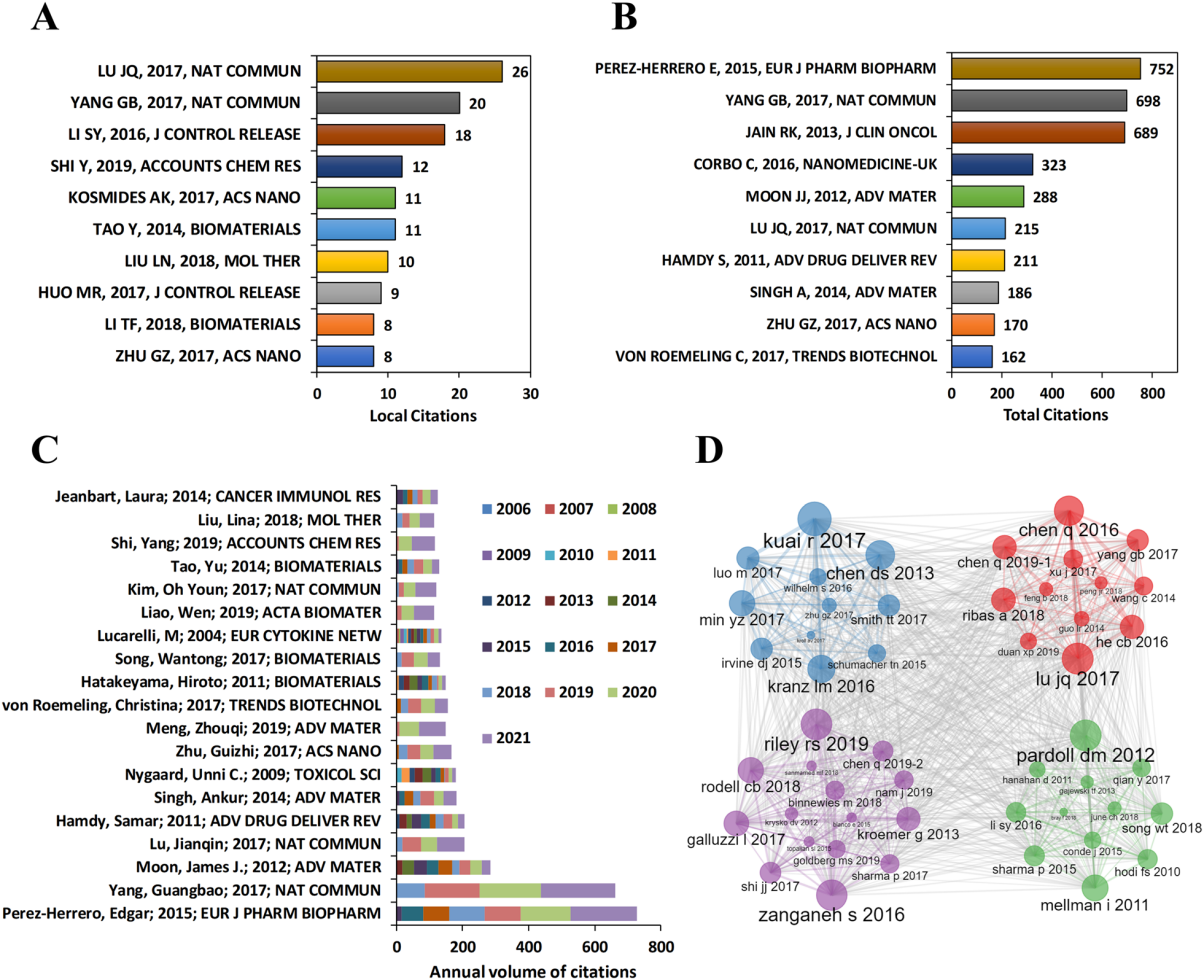
Correlation analysis of key papers. **A** The top ten papers cited by local literature. **B** The top ten papers with the highest total citations. **C** Annual citation accumulation chart of the top 20 papers with total citations. **D** Co-citation network of key papers

The application of nanomaterials in tumor immunotherapy has received extensive attention and citations. Among them, Pérez-Herrero E [28] summarized the advantages and limitations of many nanocarriers loaded with different chemotherapeutic drugs in tumor treatment. The study was cited 752 times in total (Fig. 8B) and 20 times in local literature (Fig. 8A). At the same time, the number of annual citations of this research have continued to grow in recent years (Fig. 8C). Yang G et al. [29] developed a Hollow MnO_2_-based nano-platform H-MnO_2_-PEG/C&D combined with anti-PD-L1, which can activate tumor immunity in mice and significantly inhibit primary tumors and metastatic tumors. The paper has been cited 698 times in total (Fig. 8B), 224 times last year alone, and the degree of attention has increased year by year (Fig. 8C).

Lu J et al. [30] designed a nano-platform OX/IND-MSNP, in which phospholipid bilayer-wrapped mesoporous silica nanoparticles were simultaneously loaded with oxaliplatin and immunostimulatory drugs. This nanoparticle could effectively induce tumor immunogenic cell death (ICD) and trigger the antigen presentation of dendritic cells, further inducing the activation of T cells and tumor immune memory. Lu J's paper was cited 26 times (Fig. 8A). Li SY et al. [31] constructed nanoparticles to deliver CTLA-4 siRNA (NPsiCTLA-4) and showed the ability of this siRNA delivery system to enter T cells both in vitro and in vivo, eliminating the immunosuppression in the tumor microenvironment. Li SY's paper was cited 18 times (Fig. 8A). In addition, there were also rich citation relationships between these key literatures (Fig. 8D).

In addition to the articles mentioned above, the following papers also ranked in the top ten citations in this field. Jain RK [32] found that the tumor-associated hematological and lymphatic vasculature, fibroblasts, immune cells, and extracellular matrix were abnormal, which together created a hostile tumor microenvironment. However, vascular normalization can convert the immunosuppressive tumor microenvironment into an immunoactivated tumor microenvironment, and improve the efficacy of immunotherapy via increasing blood flow and oxygenation. Corbo C et al. [33] observed that nanomaterials interacted with biological components and surrounded with a protein corona (PC) while be injected in physiological environments such as blood. This can trigger an immune response and affect the toxicity and targeting ability of the NP. Moon JJ et al. [34] reviewed the advanced findings of the nanoparticle developments for immunotherapy and diagnosis. Nanomaterials used in the tumor microenvironment or in systemic lymph nodes showed satisfying potential. Moreover, strategies to actively target cancer therapeutic agents to the tumor microenvironment using immune cells themselves as delivery vehicles were also very interesting. Hamdy S et al. [35] reviewed the applications of poly (D, L-lactic-co-glycolic acid) nanoparticles (PLGA-NPs) in cancer vaccine delivery systems. PLGA-NPs containing antigens or immunostimulatory molecules can not only actively target DC, but also rescue impaired DC from tumor-induced immune suppression. Singh A et al. [36] raised an emerging immunomodulation idea based on hydrogel and scaffold, which can be perfectly applied in a variety of tumors. In addition, hydrogels and stents can also perform well in diseases other than the tumors, such as chronic infections and autoimmune diseases. Zhu G et al. [37] reviewed the vaccines for cancer immunotherapy by synthetizing nanoparticles or naturally derived nanoparticles. Nanovaccines can effectively co-deliver immune-activating adjuvants and multiepitope antigens into lymphoid organs and antigen-presenting cells, fine-tuning the intracellular release and cross-expression of the antigen by nano vaccine engineering. von Roemeling C et al. [38] discovered that as immunotherapy became increasingly important in clinical oncology, the strategies utilizing the interactions between nanomaterials and various components of the immune system provided possibilities for exploring novel immune adjuvants to exert enhanced antitumor effects.

### Correlation analysis of impact factor

**Fig. 9:**
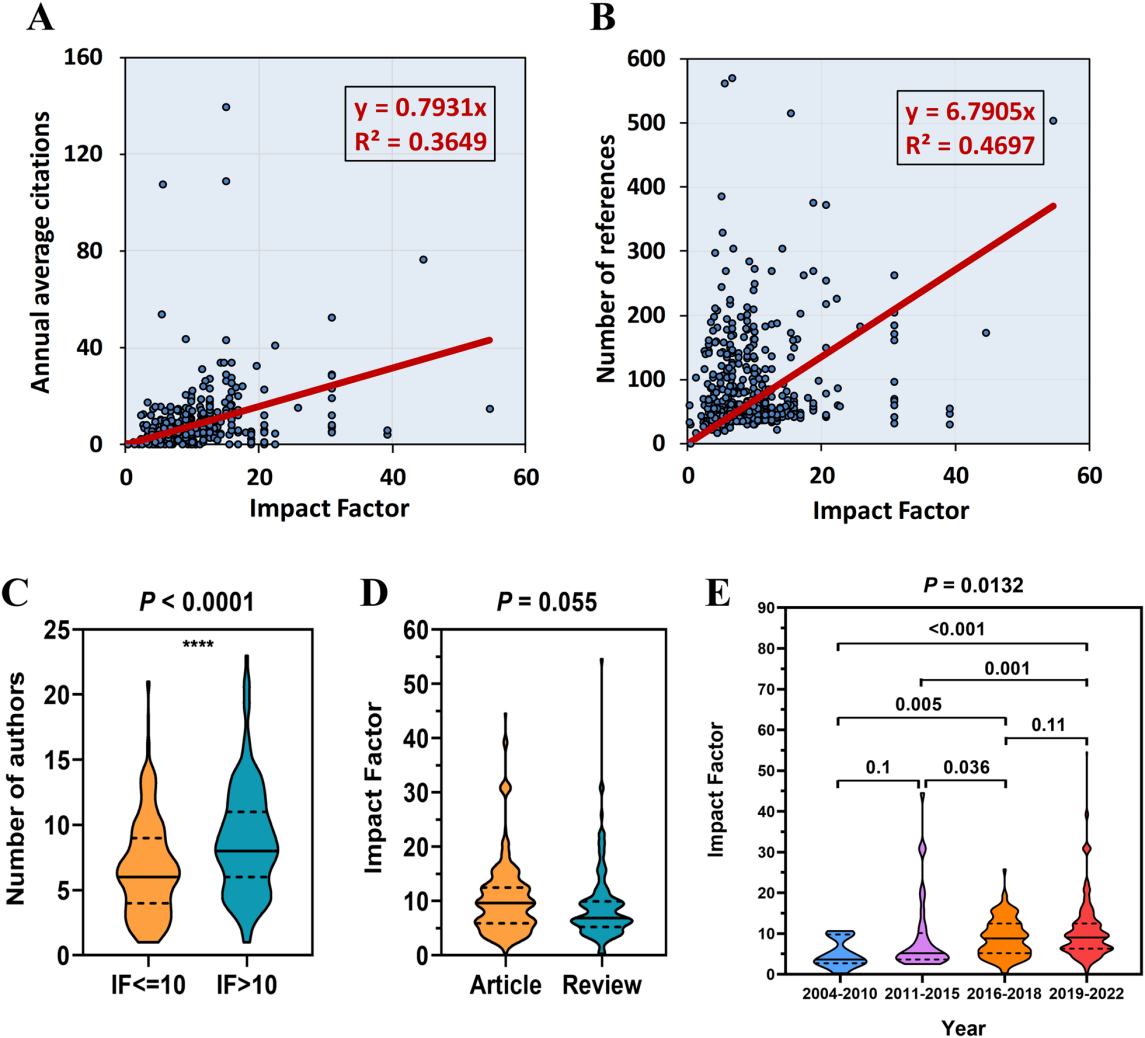
Correlation analysis of impact factor. The impact factor is positively correlated with the annual average number of citations (**A**) and the number of references (**B**). **C** Papers with an impact factor greater than ten have more authors. **D** Differences in impact factor between article and review. **E** The differences in impact factor of papers in different time periods

Correlation analysis showed that the impact factor of a study was positively correlated with the number of citations (Fig. 9A) and the number of references (Fig. 9B). The average number of authors of papers with an impact factor above 10 was significantly larger than that of papers with an impact factor of less than 10 (Fig. 9C). The average impact factor of the experimental literature (IF = 10.36) was greater than that of the review literature (IF = 9.04), however there was no statistical difference between the two (P = 0.055) (Fig. 9D). The impact factor of articles published in recent years has improved significantly, as compared with that before 2015 (Fig. 9E), and this was also considered to be related to the overall increase in impact factor.

## Discussion

In the early twenty-first century, researches applying nanomaterials in tumor immunotherapy emerged gradually. Initially, this novel idea failed to draw much attention, and the average annual number of relevant publications was not more than 10. Nevertheless, 2015 turned out to be a turning point. With researchers' realization of the promising potential that nanomaterials have on facilitating tumor immunotherapy efficacy, this field soon became hot and be further excavated by researchers. It's known that the number of published papers can be regarded as the most important indicator of whether and when a field becomes a research hotspot. The number of publications in this research field in 2021 was 126, exhibiting in the highest number of publications on this subject in a year. Moreover, the annual growth rate of relevant publications from 2004 to 2022 was found to be 16.85%. Among the 443 publications under study, the international cooperation accounted for 28.67%. The top six countries with the largest number of publications were China, the United States, South Korea, Iran, Japan and India. China's publication volume of 213 articles far exceeded that of other countries, but its citation rate was not optimistic. Notably, the United States just followed China in the number of publications but its research results were highly recognized in the peer field. The top five institutions in terms of publication volume worldwide were Sichuan University, Taipei Medical University, Wuhan University, Nanjing University, and North Carolina State University. This suggests that the recognition of antitumor nano-immunotherapy has continued to increase in recent years. The authors speculated that the number of the articles involving in antitumor nano-immunotherapy would persistently increase. Additionally, it is believed that various countries would tightly cooperate and make progress in this field.

The majority of the researches related with antitumor nano-immunotherapy have been published in the Journal of Controlled Release (2021 IF = 11.467), Biomaterials (2021 IF = 15.304), Acta Biomaterialia (2021 IF = 10.633), Theranostics (2021 IF = 11.6), Advanced Materials (2021 IF = 32.086) and ACS Nano (2021 IF = 18.027). All threse journals had the impact factor above 10. Furthermore, the top 20 journals in publication volume belonged to Q1 division (2021 JIF quartile), demonstrating that related researches were of high quality and generally recognized by top journals.

Keywords analysis revealed that nanoparticles, dendritic cells, delivery, cancer, T cells, immunotherapy, photodynamic therapy, immune checkpoint blockade, chemotherapy, tumor microenvironment, and immune response were steering the research filed. A significant correlations existed between the keywords immunotherapy and immune checkpoint blockade, photodynamic therapy, photothermal therapy, T cells, tumor-associated macrophages, tumor microenvironment and immunogenic cell death, with correlation coefficient > 0.4. Thus, it can be inferred that the key therapies for antitumor nano-immunotherapy mainly consisted of immune checkpoint blockade, photodynamic therapy, photothermal therapy and vaccine. Immune cells (including dendritic cells, T cells, macrophages, etc.) in the tumor microenvironment were modulated to exert stronger injuring effects on tumors in situ or more effective immune clearance effects on metastatic lesions. In addition, there were differences in the focus of the studies in different years. The main keywords in 2019 included dendritic cells, drug-delivery, regulatory T cells, etc. The main keywords in 2020 consisted of delivery, T cells, etc., whereas keywords in 2021 comprised of immunogenic cell-death, tumor-associated macrophages, targeted delivery, natural killer cells, hypoxia, antitumor-activity, antibody, etc.

Emerging evidence proved that dendritic cells played an indispensable role in antitumor nano-immunotherapy. It has now been established that the tumor cell death in the primary site can release tumor-associated antigens (TAAs) [39]. Dendritic cells are capable of capturing these antigens, and then present these antigens to the T cell receptor via a major histocompatibility complex (MHC) after migrating to immune organs such as spleen or lymph nodes. Ultimately, T cell-mediated long-term tumor immune are successfully triggered [40]. According to relevant studies in this filed, the key steps in the immune network could be summarized into 3 points as follows [41]: (1) In the killed tumor cells, calreticulin is transferred from the endoplasmic reticulum to the cell surface, which strongly attracts dendritic cells, further inducing phagocytosis of dendritic cells. (2) Release of high mobility group box 1 (HMGB1) activates dendritic cells mediated by toll-like receptor 4 (TLR-4). (3) Release of ATP stimulates P2X7 purinergic receptors on dendritic cells, triggering inflammasome, IL-1β secretion and CD8^+^ T cell priming. Although dendritic cells have been regarded as core theme with high maturity (Motor Themes quadrant), more researches are still needed to further seek underlying core mechanisms.

Furthermore, the delivery of nanomaterials remains a key issue and urgent to be solved in this field [2]. Currently, nanomaterials are generally delivered into tumor tissues through the EPR effect, which is defined as passive drug delivery. The size, shape, and surface charge of nanoparticles are vital factors for the efficiency of drug delivery systems [42, 43]. However, the efficacy and safety of the EPR effect have been controversial in recent years [44, 45]. Recent statistical research revealed that merely 0.76% of the intravenous nanomaterials smoothly reached solid tumors [46]. Notably, active targeting has shown effective effects on ameliorating intracellular uptake to a certain extent. Nonetheless, limited permeability of nanomaterials in tumor tissues turned out to be an unsolved problem in the process of active targeting [47]. Researches have shown that active targeting performs better in hematological cancers in which barrier to systemic circulation is relatively small [48]. A study by Setyawati et al. [49] identified a novel form of endothelial leakage, termed nanomaterial induced endothelial leakage (NanoEL). NanoEL-induced endothelial leakage depends on the disruption of vascular endothelial-cadherin (VE-cadherin), coupled with actin remodeling and cell contraction, to expand the intercellular space. Studies have indicated that TiO_2_, Au and SiO_2_ nanoparticles have significant effect on inducing the leakage of breast cancer endothelial cells [50]. Compared with the EPR effect relied on abnormal angiogenesis in mature solid tumors, nanomaterials can induce NanoEL effect by virtue of their own inherent capabilities. It can therefore be conferred that well-designed nanomaterials are capable of actively inducing leakage of vascular endothelial cells to cross blood vessels and accumulating substantially in tumor tissues, independent of tumor type and stage. However, there are series of side effects of NanoEL effect-induced vascular endothelial leakage: facilitating tumor circulatory metastasis, aggravating bacterial infection, promoting edema and thrombus formation, etc. In summary, the delivery of nanomaterials has always been a momentous part in nanomedicine field, which deserves deeper exploration.

The evolution of keywords reflected that the application of nanomaterials in immunotherapy underwent a transformation from simple into complex, phenotype into mechanism. For instance, early researches mainly concentrated on the tumor-killing effects of the material. Now we tend to pay more attention to the targeted delivery of nanomaterials, the synergistic effects of multiple anti-tumor therapies, the regulation of nanomaterials on the tumor microenvironment, and the internal mechanisms of tumor immunity. We ultimately summarize and list three mainstreams for the application of nanomaterials to tumor immunity: (1) Targeting tumor cells [39, 41]: Nanomaterials induce ICD and further release TAAs. As an important trigger and enhancer of anti-tumor immunity, nanomaterials facilitate the antigen presentation of APC. ICD can be induced by certain types of chemotherapeutic drugs (such as doxorubicin, oxaliplatin, cyclophosphamide and so on), as well as by radiation therapy, photodynamic/photothermal therapy, and other methods. (2) Targeting TIME [51, 52]: Immunosuppressive pathways and mediators are always upregulated in TIME. For example, increased infiltration of immunosuppressive cells including regulatory T cells (Tregs), myeloid-derived suppressor cells (MDSC) and M2 macrophages have been detected. Soluble inhibitors such as indoleamine 2,3 dioxygenase (IDO), transforming growth factor-beta (TGF-beta) are also increased. Nanomaterials reverse the immunosuppressive TIME and regulate the infiltration, proliferation, maturation, and activation of T cells to further polish up the immunotherapy efficacy. (3) Targeting the peripheral immune system [53, 54]: Nanomaterials promote anti-tumor immune responses through enhancing antigen presentation and generation of cytotoxic T cells in secondary lymphoid organs (such as lymph nodes and spleen), as well as modulating and augmenting peripheral effector immune cell populations.

### Supplementary Information

**Additional file 1: Figure S1.** Country scientific production.

**Additional file 2: Figure S2.** The 6 countries that contributed the most.

**Additional file 3: Figure S3.** World collaboration map.

**Additional file 4: Figure S4.** The keywords density map for different time periods. **A** 2004-2016, **B** 2017-2018, **C** 2019-2020, **D** 2021-2022.

**Additional file 5: Figure S5.** Correlation heat map between keywords.

**Additional file 6: Table S1.** Characteristic information form of all included papers.

**Additional file 7: Table S2.** Publication status of papers in all countries. **Table S3.** The contribution value of all countries. **Table S4.** Institutions that have published more than one related paper.

## Data Availability

The original contributions presented in the study are included in the article/Supplementary Material. Further inquiries can be directed to the corresponding authors.
