# Supplementary figures and images for: Forefronts and hotspots evolution of the nanomaterial application in anti-tumor immunotherapy: a scientometric analysis
Source: J Nanobiotechnology. 2024 Jan 13;22:30. doi: 10.1186/s12951-023-02278-3 (PMC10788038; doi:10.1186/s12951-023-02278-3)

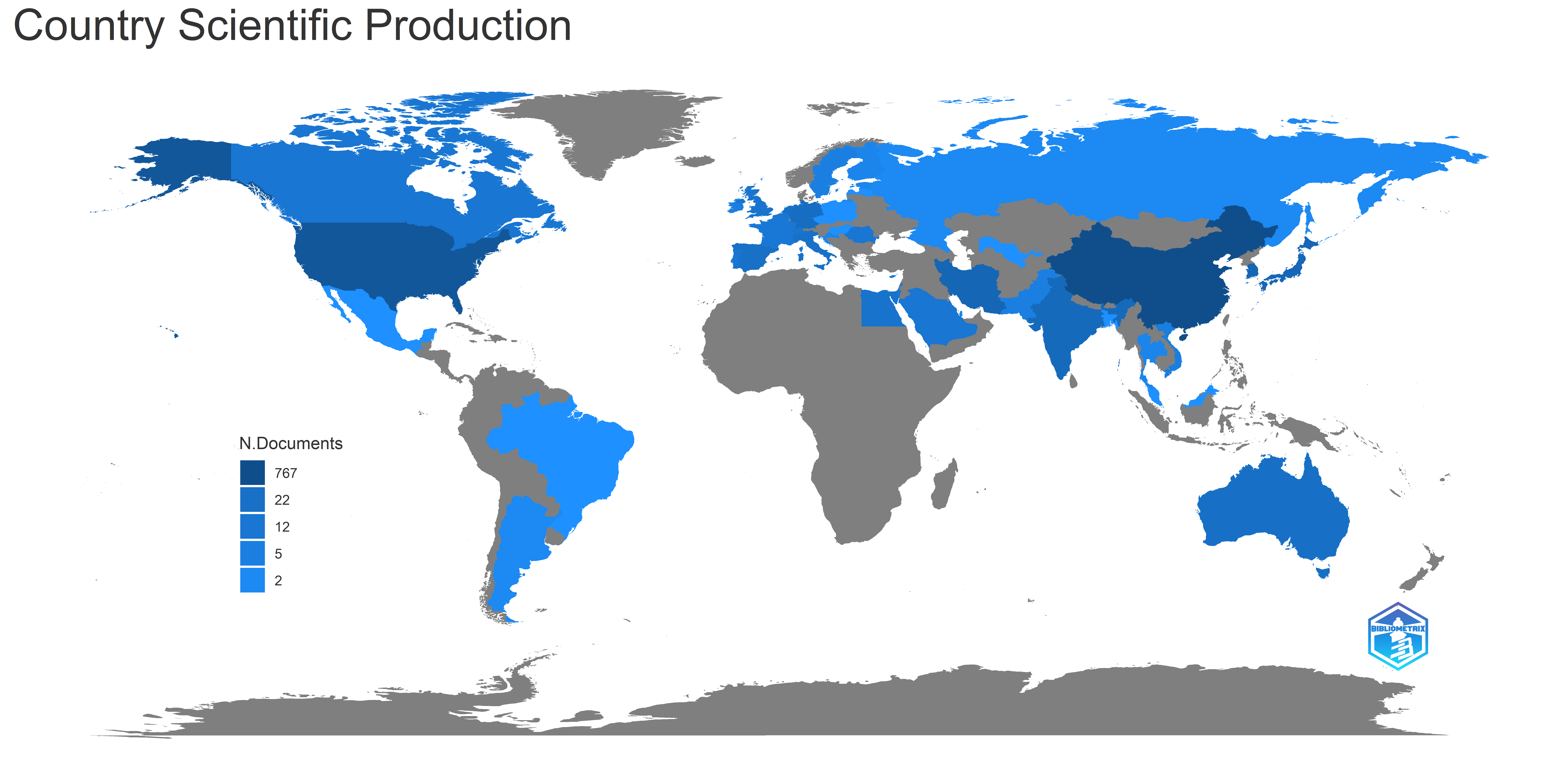

Supplement: Supplementary file 1 — Additional file 1: Figure S1. Country scientific production. [file 12951_2023_2278_MOESM1_ESM.tif]

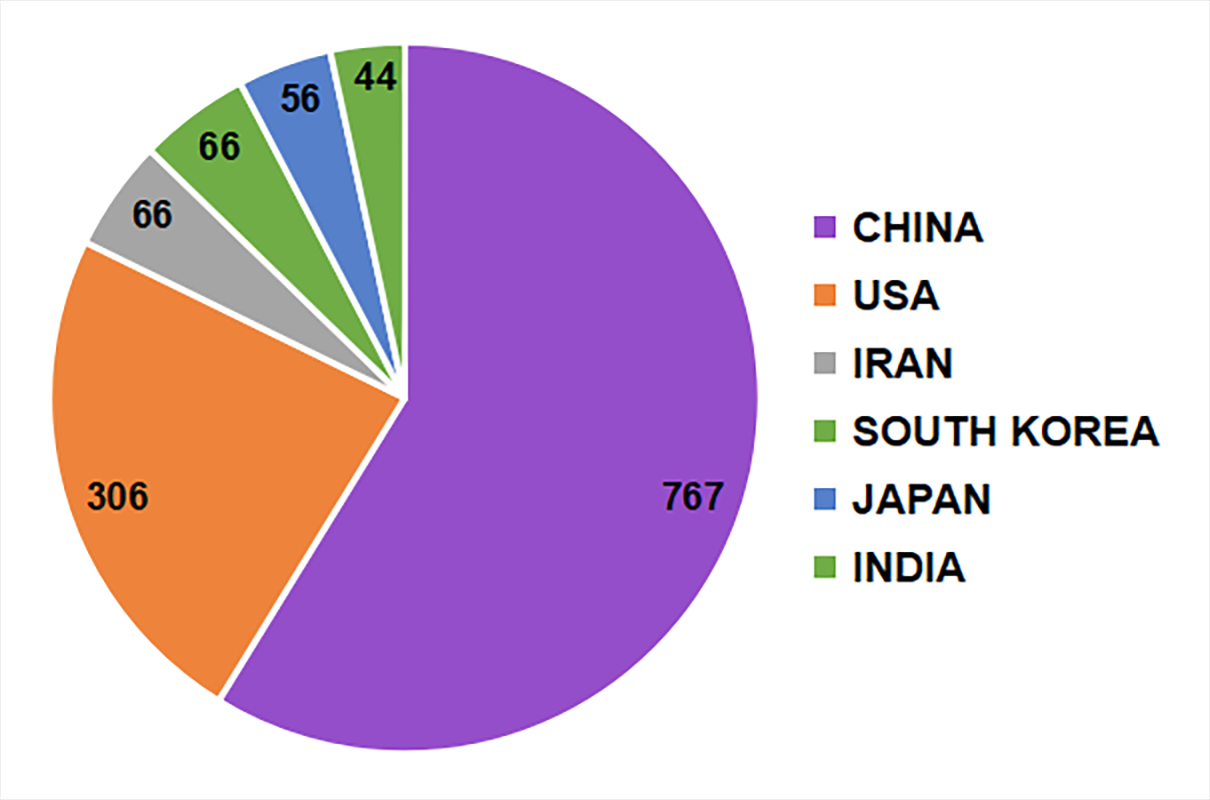

Supplement: Supplementary file 2 — Additional file 2: Figure S2. The 6 countries that contributed the most. [file 12951_2023_2278_MOESM2_ESM.tif]

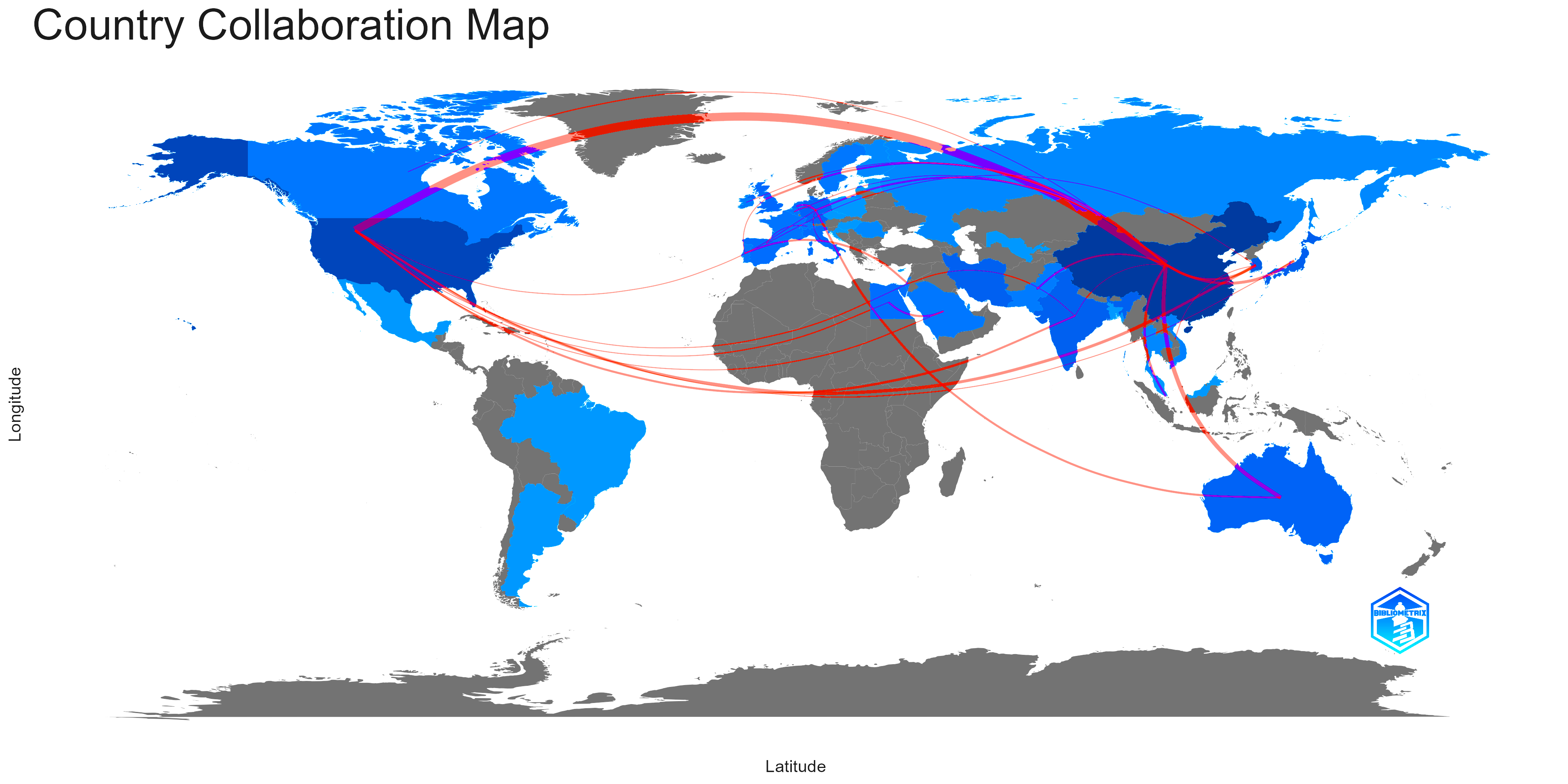

Supplement: Supplementary file 3 — Additional file 3: Figure S3. World collaboration map. [file 12951_2023_2278_MOESM3_ESM.tif]

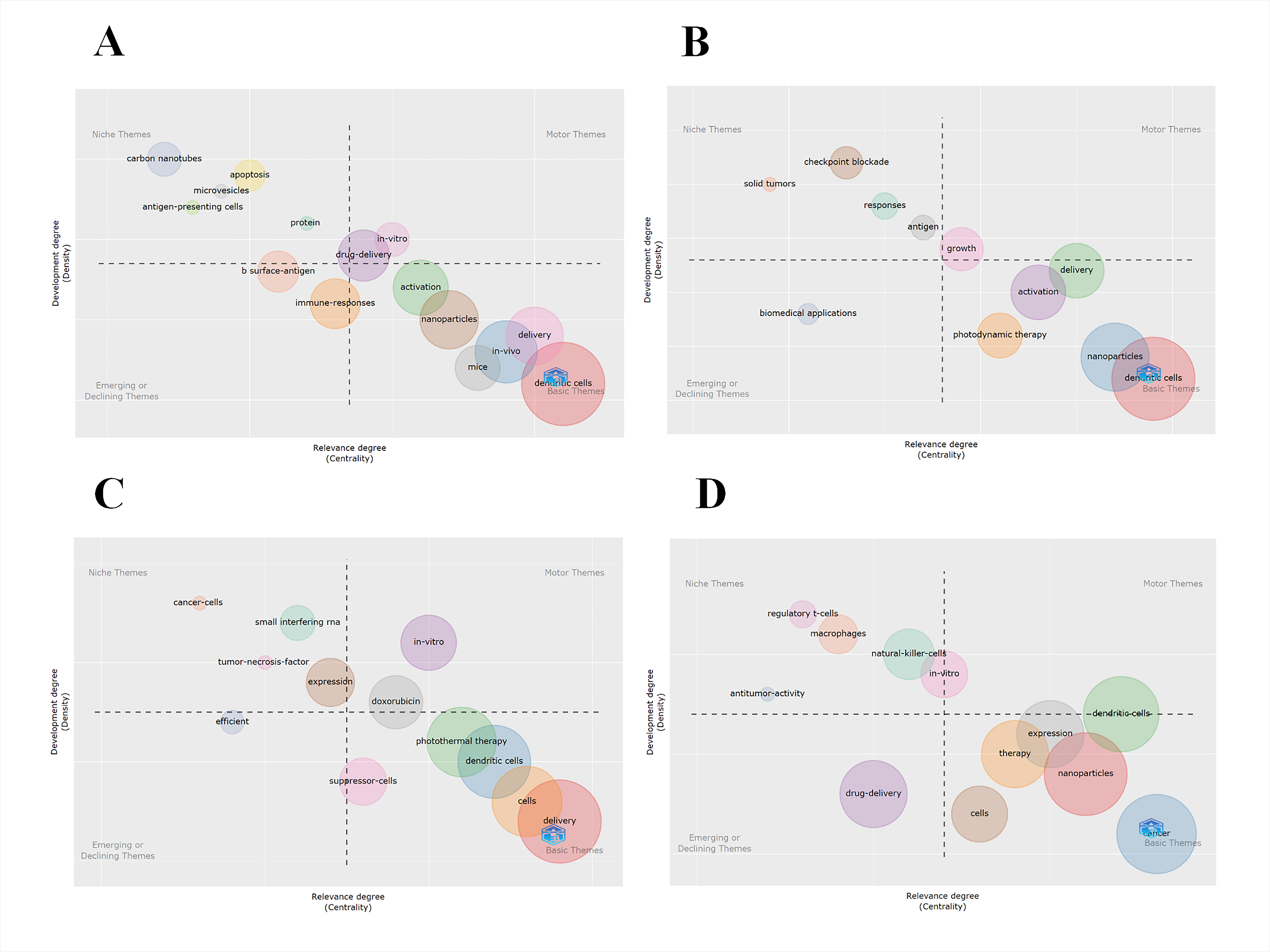

Supplement: Supplementary file 4 — Additional file 4: Figure S4. The keywords density map for different time periods. A 2004-2016, B 2017-2018, C 2019-2020, D 2021-2022. [file 12951_2023_2278_MOESM4_ESM.tif]

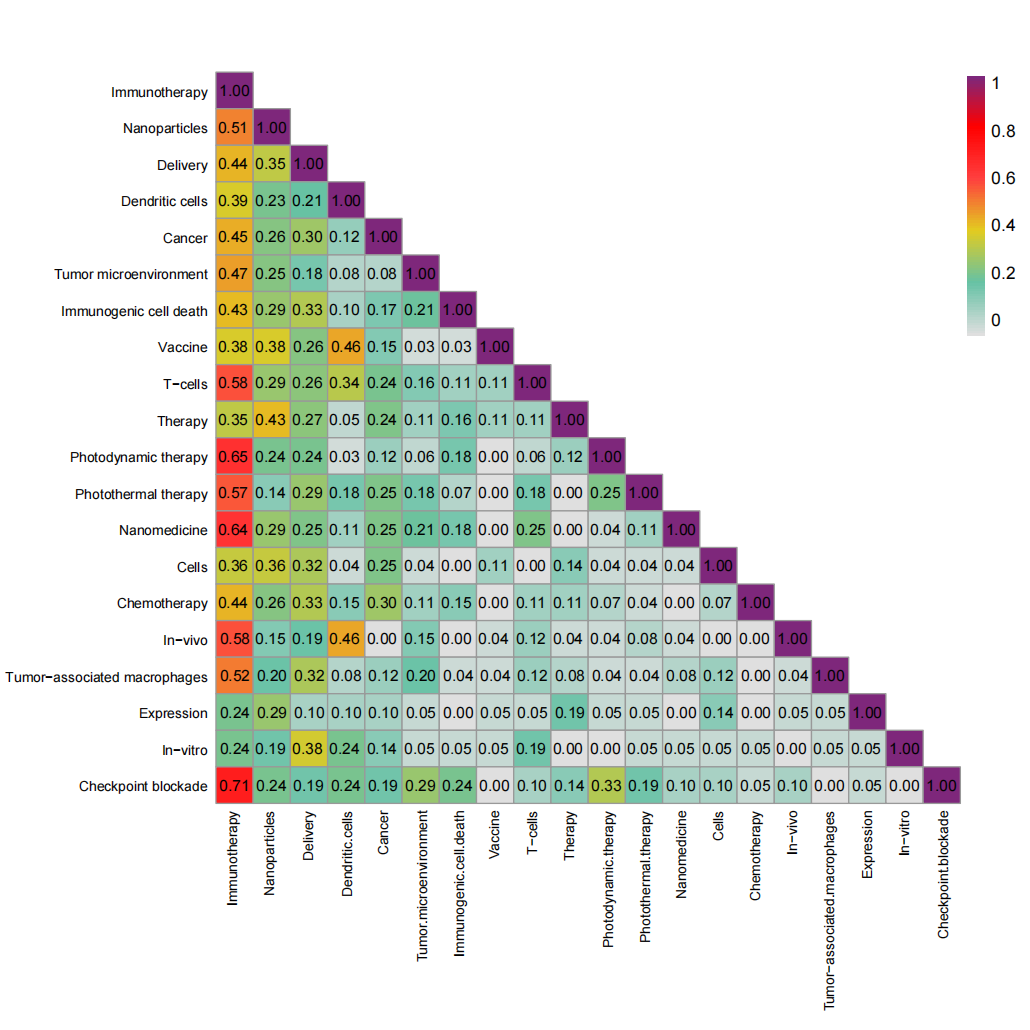

Supplement: Supplementary file 5 — Additional file 5: Figure S5. Correlation heat map between keywords. [file 12951_2023_2278_MOESM5_ESM.tif]
